# Cervical and Endometrial Cancer Incidence in the Female Population from the Bryansk Region Living in Conditions of Chemical, Radioactive and Combined Environmental Contamination (2000–2020)

**DOI:** 10.3390/life12101488

**Published:** 2022-09-25

**Authors:** Anton V. Korsakov, Anna E. Kryukova, Vladislav P. Troshin, Olga Yu. Milushkina, Dmitry G. Lagerev

**Affiliations:** 1 Department of Disaster Medicine, Faculty of Medicine, Pirogov Russian National Research Medical University (Pirogov Medical University), 117997 Moscow, Russia; 2 Department of Technosphere Safety, Bryansk State Technical University, 241035 Bryansk, Russia; 3 Department of Hygiene, Faculty of Pediatrics, Pirogov Russian National Research Medical University (Pirogov Medical University), 117997 Moscow, Russia; 4 Department of Computer Science and Software, Bryansk State Technical University, 241035 Bryansk, Russia

**Keywords:** environmental pollution, environmental assessment, environmental health, cervical cancer, endometrial cancer, Chernobyl accident, radioactive contamination, chemical pollution, combined contamination, Cesium-137, Strontium-90, average annual effective doses, correlation analysis, regression analysis, relative risk, Bryansk region

## Abstract

At the end of 36 years after the Chernobyl disaster, about 5 million people still live in the radioactively contaminated territories of Russia, Ukraine, and Belarus, and the density of radioactive contamination by Cesium-137 and Strontium-90 will remain radiologically significant for decades. We assessed cervical and endometrial cancer primary incidence (new cases) in the female population from the Bryansk region living in conditions of chemical, radioactive, and combined environmental contamination for 2000–2020. We found a significant increase in the long-term trend in the primary incidence of cervical and endometrial cancer in all the studied groups, regardless of the environmental conditions of residence (*p* < 0.00001). We did not find statistically significant differences in the incidence of cervical and endometrial cancer in women, regardless of the level of chemical, radioactive, and combined environmental contamination. However, women living in environmentally unfavorable areas (in total, in the territories of chemical, radioactive, and combined contamination) are statistically significantly more likely to develop endometrial cancer in terms of relative risk compared to environmentally safe (control) areas (RR 1.17 (1.08–1.27)). No such pattern was found for cervix cancer. It should be noted, since environmentally safe (control) areas have a certain level of contamination (albeit low), RR is underestimated.

## 1. Introduction

According to the International Agency for Research on Cancer (IARC) GLOBOCAN 2020 [1], 19.3 million new cases of cancers and almost 10 million deaths from them have been registered in the world. According to IARC forecasts, 28.4 million cases of cancers are expected to be detected in 2040, which is 47% more than in 2020. Cervical cancer is the fourth most commonly diagnosed cancer and the fourth leading cause of cancer death in women. In 2020, there were 604,000 new cases and 342,000 deaths worldwide [1]. Endometrial cancer is the sixth most commonly diagnosed cancer in women (417,000 new cases and 97,000 deaths in 2020) [1].

According to the Russian Research Institute of Oncology named after P.A. Herzen [2] in the Russian Federation in 2020, endometrial cancer ranks second (7.0% of patients), and cervical cancer is the seventh (4.7% of patients) most common in the structure of all malignant neoplasms. It should be noted that in 2020, among patients with newly diagnosed cervical cancer, 33.6% of women were diagnosed with stages III-IV [2], which worsens the prognosis of effective treatment and quality of life.

A number of studies reveal a statistically significant relationship between the risk of cancers of the female reproductive system and an increase in the level of radioactive contamination [3,4,5,6,7,8,9,10,11,12] and chemical pollution [13,14,15,16,17,18,19,20,21,22] of the environment.

At the end of 36 years after the Chernobyl disaster, about 5 million people still live in the radioactively contaminated territories of Russia, Ukraine, and Belarus, and the density of radioactive contamination by Cesium-137 (^137^Cs) and Strontium-90 (^90^Sr) will remain radiologically significant for decades [3,23].

Moreover, 36 years after the Chernobyl disaster, 309 thousand people still live in the radioactively contaminated territories of the Bryansk region, 60% of whom are women [24]. The geographical distance of the city of Bryansk from Chernobyl is 221 miles in a straight line, 272 miles along the highway.

Regular radioecological monitoring in the Bryansk region indicates that the density of soil contamination by ^137^Cs and ^90^Sr in the southwestern territories (SWT) significantly exceeds the established reference levels [25], while the accumulated doses of public exposure 36 years after the Chernobyl accident fluctuate in the range from units to hundreds of mSv [26].

According to official data [27], in recent years in the Bryansk region, there has been an increase in emissions of air pollutants, mainly carbon monoxide (CO) and volatile organic compounds (VOCs).

It should be noted that in some territories of the Bryansk region, the population is exposed to the combined influence of radioactive and chemical environmental contamination [28,29,30].

Thus, it was found that the combination of chemical and radioactive contamination led to a significantly higher incidence of multiple congenital malformations compared to regions where there is only one pollutant [28]. These results suggest an additive and potentially synergistic effect of radioactive and chemical agents on the incidence of multiple congenital malformations.

Thus, a sharp increase in the mutation process resulting from environmental contamination poses a threat to the genetic safety of all living things [31].

In this regard, the study of the health status of the female population living in environmentally unfavorable conditions is very relevant. To this end, we analyzed the dynamics of the cervical and endometrial cancer incidence in the female population from the Bryansk region, living in conditions of chemical, radioactive, and combined environmental contamination over a multi-year period (2000–2020).

## 2. Materials and Methods

We performed an extensive analysis of the hygienic state of the environment during 2000–2019 in all cities and districts of the Bryansk region, taking into account the levels of radiation (due to the Chernobyl disaster), chemical (due to air pollution), and combined radiation-chemical contamination. Then, we carried out a comparative assessment of the dynamics of the incidence of cervical and endometrial cancer in the female population in the study areas differing in environmental conditions for 2000–2019. Primary incidence (in the following the term “incidence” is used) was estimated as the number of new cases of cervical and endometrial cancer (“incidence rate”).

For the density of radioactive contamination of the territories by ^137^Cs and ^90^Sr as a result of the Chernobyl disaster were determined for 2000–2019 according to [25,32]. This was determined as the average annual values in each settlement of the Bryansk region for a twenty-year period according to radioecological monitoring data (2000–2019). The average annual effective dose (AAED_90_) from external and internal radiation exposure of residents in the radioactively contaminated areas of the Bryansk region of exposure were determined also for 2000–2019 according to the data [26,32]. External and internal radiation doses of residents in radioactively contaminated areas of the Bryansk region were determined using the following for the baseline data: (1) measurement results of the ^137^Cs and ^90^Sr per unit activity in samples of locally originating milk, potato, and mushrooms; (2) data with respect to soil groups and types dominant in the settlements or communal farms; (3) data with respect to a housing stock structure in the settlements; and (4) official data on densities of soil contamination by ^137^Cs and ^90^Sr in the settlements.

According to the data of the Department of the Federal State Statistics Service for the Bryansk region for 2000–2019 [24], the level of chemical pollution was estimated—reports of emissions of pollutants into the atmosphere from stationary sources, tons per year. We have identified the main air pollutants: carbon monoxide (CO), nitrogen oxides (NO_x_), sulfur dioxide (SO_2_), and VOCs (including formaldehyde, benzene, benz(a)pyrene, styrene, pyridine, vinyl chloride, acrolein, and phenol).

The recalculation of emissions of chemicals into the atmospheric air (tons/year) was made for the area of the region (km^2^) in (g/m^2^) [24].

We used the depersonalized data of official state statistics aggregated by settlements of the Bryansk region without indicating the personal data of the patients by the incidence of cervical and endometrium cancer in woman over 18 years of age for 2000–2020 according to the data of the Bryansk regional oncological dispensary (Cancer registry of the Bryansk region) [33]. These data reflect the natural retrospective process of oncological diseases in the cities and districts of the Bryansk region. Experiments were not performed and informed consent from patients was not required. In total, 2325 cases of cervical cancer and 4513 cases of endometrial cancer were registered in 2000–2020. Including in environmentally safe territories (control) 422 cases of cervical cancer and 698 cases of endometrial cancer were detected, in the territories of chemical pollution 1433 and 2960 cases, in the territories of radioactive contamination 182 and 307 cases, and in the territories of combined contamination 288 and 548 cases, respectively [33].

The recalculation of the absolute values of the incidence of cervical and endometrial cancer was carried out per 100,000 women over 18 years of age in cities and districts of the Bryansk region (the observation period for calculating the incidence is one year) [24].

Using the tools of the Stata SE 14.2 package, a statistical analysis of the obtained data was carried out. We used the sample mean (M) and the standard error of the mean (SEM). Using the Shapiro–Wilk W test, the normality of data distribution was determined. It showed that the distribution is not normal, both for all pollutants, and for ^137^Cs, ^90^Sr, and cervical and endometrial cancer. Therefore, to assess the relationship between the level of chemical and radioactive contamination and the incidence of cervical and endometrial cancer, we used the Spearman rank correlation test. The Wilcoxon rank-sum (Mann–Whitney) test was applied to test the statistical significance of differences [34].

We calculated the relative risk (RR) for the retrospective cohort study of the incidence of cervical and endometrial cancer in areas with different levels of radiation, chemical, and combined environmental contamination.

We calculated the Poisson regression of the level of incidence of cervical and endometrial cancer [35]. Moreover, the forecast of the level of incidence of cervical and endometrial cancer was calculated. We took the data for 2000–2019, calculated the Poisson regression forecast for 2020, and compared the actual values for 2020 with the predicted ones. The presented forecast allows us to assess how the actual values of the incidence rate in the context of the COVID-19 pandemic differ from the predicted ones.

## 3. Results

We have ranked the cities and districts of the Bryansk region depending on the density of radioactive contamination by ^137^Cs and ^90^Sr, on the levels of chemical pollution of atmospheric air by CO, NO_x_, SO_2_, and VOCs, as well as the incidence of cervical and endometrial cancer in the female population (Table 1).

The levels of chemical pollution by leading gaseous substances and the density of radioactive contamination by ^137^Cs and ^90^Sr fluctuate over a wide range in the Bryansk region (Table 1). By chemical pollution of atmospheric air (g/m^2^) from 13 to 32190, of which: for VOCs from 0 to 5217, NO_x_ from 6 to 10,886, SO_2_ from 0 to 2617, and CO from 7 to 13470. For ^90^Sr—from 0.4 to 16.3 kBq/m^2^, for ^137^Cs—from 4.4 to 460.6 kBq/m^2^.

The values of the average annual effective dose (AAED_90_) from the Chernobyl component in the group of environmentally safe areas (control) and areas of chemical pollution do not exceed 0.1 mSv per year, while in the group of areas of radioactive and combined contamination, AAED_90_ averages 1.2 mSv per year. The maximum values in 2020 were recorded in the village of Barsuki, Krasnogorsk district, amounting to 5.6 mSv per year. The density of radioactive contamination is much less than the established standards (criteria for classifying territories as zones of radioactive contamination) for both ^137^Cs (up to 37 kBq/m^2^) and ^90^Sr (up to 5.6 kBq/m^2^) in the group of environmentally safe territories. The level of atmospheric pollution with gaseous substances ranged from 13 to 128 g/m^2^, which makes it possible to classify these territories as environmentally safe (control). The level of incidence of cervical cancer in environmentally safe areas ranges from 12.9 to 28.1. The average value over a twenty-year period was 19.7, which is 14.9% less than the all-Russian values (23.2). The level of incidence of endometrial cancer in environmentally safe areas ranges from 21.5 to 46.6. The average value for 2000–2019 was 32.3, which is 8.7% less than the all-Russian values (35.4) (Table 1). It should be noted that the incidence of cervical and endometrium cancer in the control areas is underestimated compared to the all-Russian values to the extent that the control areas are also contaminated (albeit low). Emissions of gaseous substances in chemically polluted areas exceed similar indicators of environmentally safe areas by tens, hundreds, and sometimes thousands of times, fluctuating over a wide range from 123 to 32,190 g/m^2^. At the same time, the density of contamination by long-lived radionuclides ^137^Cs varies from 4.4 to 38.4 kBq/m^2^, and ^90^Sr from 0.4 to 5.9 kBq/m^2^. The values of AAED_90_ from the Chernobyl component, as in the group of control territories, do not exceed 0.1 mSv per year. The incidence of cervical cancer varies in this group of areas from 15.8 to 25.0. The average value is 18.6, which is 19.7% less than the all-Russian values and 5.6% less than in environmentally safe areas. The incidence of endometrial cancer varies from 12.7 to 45.6. The average value is 38.2, which is 8.0% higher than the all-Russian values and 18.3% higher than in environmentally safe areas. However, the differences are not significant (Table 1).

In the areas of radioactive contamination, the density by ^137^Cs contamination exceeds the standards by 3.8–12.4 times and ranges from 139.6 to 460.6 kBq/m^2^. The density by ^90^Sr contamination reaches its maximum values in the Zlynkovsky district (16.3 kBq/m^2^), which exceeds the established norms by 2.9 times. However, in the Gordeevsky and Klintsovsky districts, the contamination level was 5.0 and 4.7 kBq/m^2^. Thus, in the radioactively contaminated areas AAED_90_ the exposure of the population varies from 0.6 to 1.9 mSv per year. At the same time, atmospheric pollution is low and comparable with the values of environmentally safe areas, ranging from 15.0 to 169 g/m^2^. We found that in the areas of radioactive contamination, the incidence of cervical cancer ranges from 12.6 to 23.3. The average value is 19.3, which is 16.7% less than the all-Russian values and practically coincides with the control regions (−2.0%). The incidence rate of endometrial cancer ranges from 18.2 to 51.3. The average value is 32.5, which is 8.1% less than the all-Russian values and practically coincides with the control regions (+0.6%) (Table 1).

In the areas of combined radiational and chemical contamination, the density of radioactive contamination by ^137^Cs, as well as in the radiation-contaminated territories, exceeds the established standards (by 1.23–12.3 times), amounting to 45.4–456.5 kBq/m^2^. The city of Novozybkov recorded the highest density of ^137^Cs contamination (456.5 kBq/m^2^). The density by ^90^Sr contamination is also exceeded and amounts to 9.7 kBq/m^2^. AAED_90_ range from 0.3 to 1.9 mSv per year. Moreover, in addition to the high level of radioactive contamination, the level of chemical pollution is 2.6–491 times higher than the values of radiation-contaminated areas, amounting to 392–7422 g/m^2^. This makes it possible to refer these areas to the category of combined contamination (Table 1). The incidence rate of cervical cancer in conditions of combined contamination varies from 14.7 to 23.6. The average value was 20.0, which is 13.6% less than the all-Russian values and practically coincides with ecologically safe territories (+1.5%). The incidence rate of endometrial cancer varies from 26.1 to 42.4. The average value is 36.9, which is 4.3% higher than the all-Russian values and 14.2% higher than in environmentally safe areas (Table 1).

The results obtained indicate the absence of statistically significant differences in the incidence of cervical and endometrial cancer in the areas of the Bryansk region, regardless of the environmental conditions of residence (*p*-values according to the Wilcoxon rank-sum (Mann–Whitney) test vary for cervical cancer from 0.61 to 0.96 and for endometrial cancer from 0.24 to 0.84) (Table 1).

The dynamics of the incidence of cervical and endometrial cancer (absolute values) in environmentally different areas of the Bryansk region in 2000–2020 is presented in Table 2. The data in Table 2 show that the number of cervical cancers in the areas of chemical pollution ranges from 29 to 108 cases per year and for endometrial cancer from 58 to 201. In the areas of radioactive contamination, the rates vary from 1 to 17 and from 5 to 30 cases. In the areas of combined contamination, the rates vary from 6 to 21 and from 10 to 44 cases, respectively. In environmentally safe areas, the rates vary from 10 to 33 and from 12 to 56 cases, respectively.

The dynamics of the level of incidence of cervical and endometrial cancer (per 100,000) in environmentally different areas of the Bryansk region in 2000–2020 is presented in Table 3. The data in Table 3 show that the incidence of cervical and endometrial cancer differs from the absolute values. So, in the areas of chemical pollution, the incidence of cervical cancer varies from 7.8 to 30.8 and for endometrial cancer from 15.5 to 57.1. In the areas of radioactive contamination, the indicators range from 2.1 to 43.5 and from 10.0 to 74.3. In the areas of combined contamination, the indicators range from 5.7 to 31.5 and from 14.2 to 63.1. In environmentally safe areas, the indicators range from 8.8 to 36.7 and from 11.2 to 56.1, respectively.

An assessment of the relative risk (RR) of the incidence of cervical cancer in areas with different levels of radiation, chemical, and combined environmental contamination in the period 2000–2019 confirmed our previously described patterns in Table 1 (Table 4).

We did not find an increased risk of cervical cancer in populations living in environmentally unfavorable areas compared with environmentally safe (control) areas. RR values vary from 0.97 to 1.03 (0.81–1.21) (Table 4). However, since control areas have a certain level of contamination (albeit low), RR is underestimated.

In contrast to the incidence of cervical cancer, the population living in environmentally unfavorable areas (in total in the areas of chemical, radioactive, and combined contamination) is statistically significantly more likely (*p* < 0.0002) to develop endometrial cancer compared to environmentally safe areas (RR 1.17 (1.08–1.27)) (Table 5). The most pronounced risk in relation to environmentally safe areas is recorded in the areas of chemical pollution (*p* < 0.00001) (RR 1.20 (1.10–1.31)) and in the areas of combined contamination (*p* < 0.01) (RR 1.16 (1.03–1.30)). At the same time, no significant patterns were found in the areas of radioactive contamination (*p* = 0.89) (RR 0.99 (0.86–1.14)) (Table 5).

We did not reveal an increased risk of endometrial cancer in the areas of radioactive contamination relative to the areas of chemical pollution (RR 0.83 (0.73–0.93)) and the areas of combined contamination and chemical pollution (RR 0.97 (0.88–1.06)). However, the population living in areas of combined contamination is more at risk of developing endometrial cancer compared to the areas of radioactive contamination (RR 1.17 (1.01–1.35)), *p* < 0.03 (Table 5).

**Figure 1:**
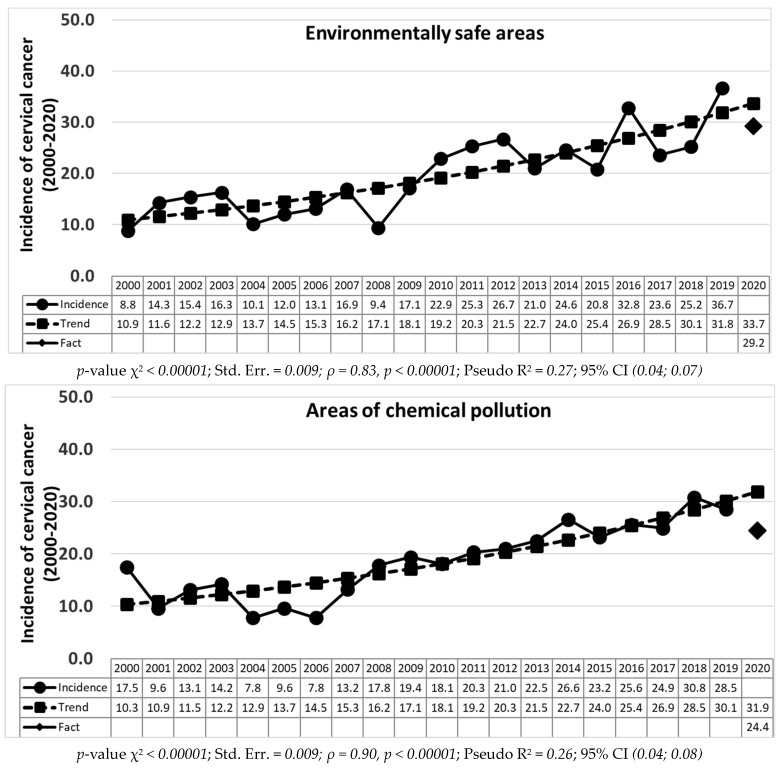

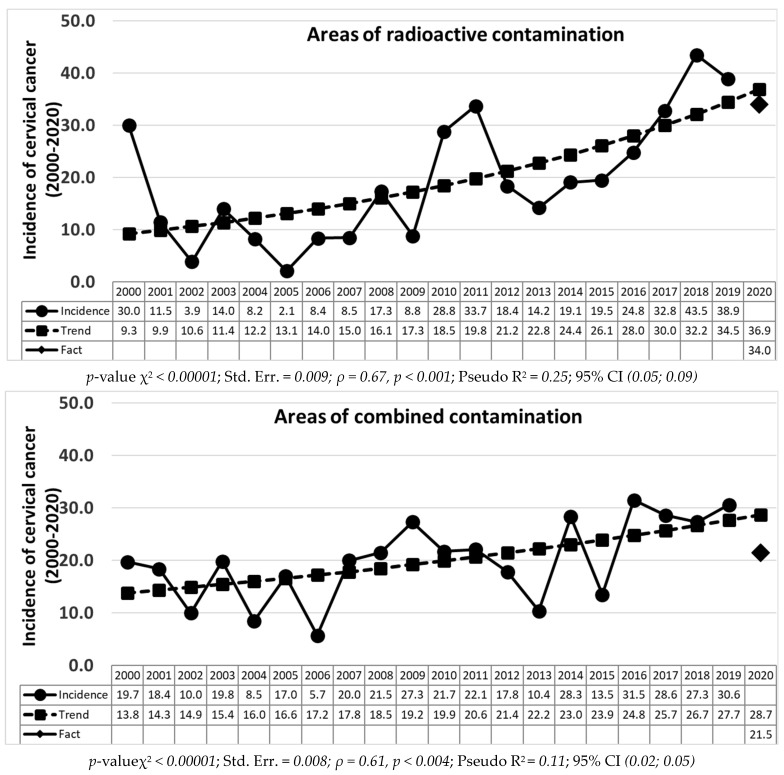
Dynamics of the incidence of cervical cancer in environmentally different areas of the Bryansk region with long-term trend lines for 2000–2019 and forecast for 2020 (per 100,000).

**Figure 2:**
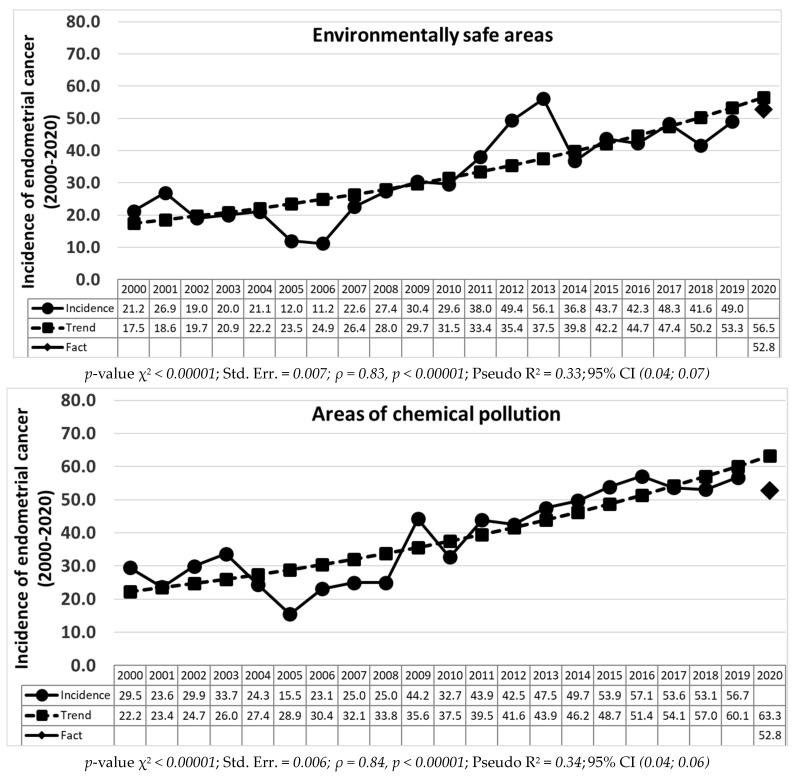

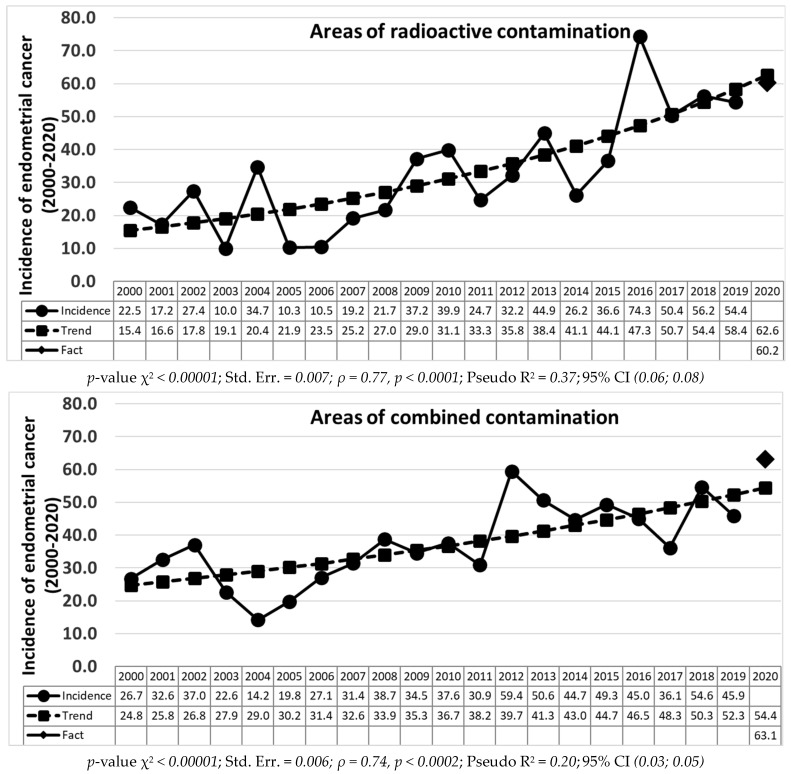
Dynamics of the incidence of endometrial cancer in environmentally different areas of the Bryansk region with long-term trend lines for 2000–2019 and forecast for 2020 (per 100,000).

Based on the calculations of the Poisson regression, a statistically significant (*p* < 0.00001) increase in the long-term trend for 2000–2019 was revealed in both the incidence of cervical cancer (Figure 1) and endometrial cancer (Figure 2) in all the areas groups, regardless of environmental living conditions.

Correlation analysis of the incidence of cervical cancer in cities and districts of the Bryansk region with the level of radiation contamination and chemical pollution of the environment (Table 6) did not reveal significant relationships both with the level of air pollution by the sum of pollutants (ρ = 0.05, *p* = 0.79), VOCs ( ρ = −0.01, *p* = 0.95), CO (ρ = 0.11, *p* = 0.57), NO_x_ (ρ = 0.09, *p* = 0.64), and SO_2_ (ρ = 0, 13, *p* = 0.50), and with the contamination density of ^137^Cs (ρ = 0.13, *p* = 0.50) and ^90^Sr (ρ = 0.19, *p* = 0.31).

No significant correlations were found for endometrial cancer, either for the sum of pollutants (ρ = 0.19, *p* = 0.32), VOCs (ρ = 0.20, *p* = 0.28), CO (ρ = 0.16, *p* = 0.38), NO_x_ (ρ = 0.18, *p* = 0.32), and SO_2_ (ρ = 0.30, *p* = 0.10), and with ^137^Cs contamination density (ρ = 0.18, *p* = 0.35) and ^90^Sr (ρ = 0.34, *p* = 0.06). However, it should be noted that the direct correlation between the incidence of endometrial cancer and the density by ^90^Sr contamination (ρ = 0.34, *p* = 0.06) is close to significance.

The forecast for the level of incidence of cervical and endometrial cancer in 2020 on average in the Bryansk region shows a decrease in real values in comparison with the forecast data by 20.7 and 10.6% (32.1 forecast in 2020 for cervical cancer and 61.1 for endometrial cancer; 25.5—real values in 2020 for cervical cancer and 54.6 for endometrial cancer). The reason for this is most likely the reorientation of the healthcare system due to the COVID-19 pandemic.

It should be noted that the decrease in real values relative to the forecast ones is uneven. Thus, the greatest decrease in cervical cancer is recorded in the areas of combined contamination by 25.1% (28.7 forecast for 2020, 21.5 real values for 2020), and a less pronounced decrease was found in the areas of radioactive contamination by 7.8% (forecast 36.9, real result 34.0). In the areas of chemical pollution, the indicators decrease by 23.6% (31.9 vs. 24.4) and in environmentally safe territories by 13.3% (33.7 vs. 29.2) (Figure 1).

The greatest decrease in endometrial cancer was found in the areas of chemical pollution by 16.6% (63.3 forecast for 2020, 52.8 real values for 2020), and a less pronounced decrease was found in the areas of radioactive contamination and environmentally safe areas by 3.9 and 6.5% (forecast 62.6 and 56.5, real result 60.2 and 52.8, respectively). It should be noted that in the areas of combined contamination, the real values of the incidence of endometrial cancer exceed the predicted values by 15.9% (forecast 54.4, real result 63.1) (Figure 2).

## 4. Discussion

As a result of the hygienic assessment of the state of the environment, we did not reveal an increased incidence of cervical and endometrial cancer in environmentally unfavorable areas in comparison with environmentally safe areas, as well as a relationship between the level of chemical and radioactive contamination with the incidence of cervical and endometrial cancer. This indicates the influence on the female reproductive system to a greater extent of endogenous factors than exogenous ones.

This work confirms our earlier research [29], which did not reveal statistically significant differences in the incidence of ovarian cancer in the female population over a long period, regardless of the environmental conditions of residence. However, the difference of our research is that women living in environmentally unfavorable areas (in total, in the territories of chemical, radioactive, and combined contamination) are statistically significantly more likely to develop endometrial cancer in terms of relative risk compared to environmentally safe (control) areas (RR 1.17 (1.08–1.27)). No such pattern was found for cervical cancer.

It should be noted that a number of studies have established a relationship between the incidence of cervical cancer and air pollution [13,15,16,17], water [14,19,20], as well as an increase in the dose of UV radiation [12]. A relationship was also found between the incidence of endometrial cancer and water pollution with cadmium [21] and with an increase in the accumulated dose of radiation due to the atomic bombings in Hiroshima and Nagasaki [7]. However, there are few works of this kind.

In [36], it was found that with an increase in the concentration of arsenic in drinking water in patients with low-grade cervical cancer with squamous cell histology, a significant dose dependence was observed, which indicates a worse prognosis of effective treatment and quality of life for women living in these territories.

There are a large number of both exogenous and endogenous risk factors for the occurrence of cancer, which are almost impossible to foresee. According to the literature [37,38,39,40,41,42,43,44,45] and WHO [46,47], among the main risk factors for the occurrence of cancer (including cervical and endometrial cancer) are tobacco smoking, alcohol consumption, human papillomaviruses, unbalanced nutrition, physical inactivity, alimentary obesity, hereditary predisposition, chemical (polycyclic aromatic hydrocarbons, dioxins, pesticides, aflatoxins, arsenic, formaldehyde, nickel, asbestos, cadmium, and many others), physical (ionizing and ultraviolet radiation), and biological (infections caused by viruses, bacteria, or parasites) environmental carcinogens.

Due to the increase in technogenic pollution of the biosphere by “global” and “eternal” pollutants, there is a steady upward trend in the incidence of cancers in the world, which is a reflection of the general trends in the increase in the “genetic burden” in human populations [31].

Analyzing the data obtained, it is necessary to emphasize the need to perform a comprehensive hygienic monitoring of the environment, depending on the level of chemical, radioactive, and combined contamination over a long period, because the influence of individual environmental factors in real conditions is always summed up and transformed (the phenomenon of synergism) [28,29,30].

It is fair to note that the mutual reinforcement and mutual suppression of different factors has been repeatedly shown in world science with the help of experiments on different test systems [47,48,49,50,51,52,53,54], but in isolated cases in real conditions of the environment [28,29,30]. This indicates the difficulty of choosing suitable research objects, identifying the relationship of factors that differ in nature and effects, and the possibility of studying statistically significant data.

Such an analysis is extremely important for assessing low-level radioactive contamination and determining the impact of associated chemical pollution in radioactively contaminated areas.

It is important to note that the quality and timeliness of diagnostic measures affect the detection of cervical and endometrial cancer [2]. Primary diagnosis of uterine pathologies is carried out on the basis of gynecological examination data, the results of smears from the cervical canal and genital tract, as well as ultrasound examination of the reproductive system. Given that the occurrence of cancers is a staged process, the diagnosis of precancerous diseases of the cervix and endometrium (dysplasia of different stages), carried out as part of screening, will lead to an interruption in this pathogenetic chain, which will affect the reduction in incidence and mortality from cervical and endometrial cancer.

The uneven nature of the decrease in real values of the incidence of cervical and endometrial cancer from the predicted ones from 3.9 to 25.1% (in one case, an excess of 15.9%), in addition to many endogenous and exogenous factors, can be explained by the different quality of medical examinations in cities and districts of the Bryansk region and the reorientation of the healthcare system due to the COVID-19 pandemic in 2020.

The limitations of our study should be noted. We analyzed the dynamics of the incidence of cervical and endometrial cancer of the female population without taking into account age groups, distribution at the stage of the disease, and histological and immunohistochemical profile.

The obtained results provide a basis for further work to understand the trends in the presence and absence of independent and combined effects of pollutants on the cancer incidence of the female reproductive system from the standpoint of assessing distant and regional metastasis, the histological and immunohistochemical profile of a particular cervical and endometrial cancer with levels of chemical, radioactive, and combined environmental contamination.

## 5. Conclusions

1. There were no statistically significant differences in the incidence of cervical and endometrial cancer in women, regardless of the level of chemical, radioactive, and combined environmental contamination.

2. There was no increased risk of cervical cancer in the female population living in environmentally unfavorable areas compared to environmentally safe (control) areas (RR values range from 0.97 to 1.03).

3. The female population living in environmentally unfavorable areas (in total in the territories of chemical, radioactive, and combined contamination) is statistically significantly more likely (*p* < 0.0002) to develop endometrial cancer compared to environmentally safe areas (RR 1.17 (1.08–1.27)).

4. No significant correlations were found between the incidence of cervical and endometrial cancer, both with the density of ^137^Cs and ^90^Sr contamination, and air pollution with gaseous substances (VOCs, SO_2_, CO, and NO_x_).

5. A statistically significant (*p* < 0.00001) increase in the long-term trend in the incidence of cervical and endometrial cancer in the period 2000–2019 was revealed in all the studied groups, regardless of the environmental conditions of residence.

6. A decrease in the incidence of cervical cancer in the Bryansk region was established by 18.0% compared to the all-Russian values, but an increase in the incidence of endometrial cancer by 3.4% compared to the all-Russian values.

7. The forecast of the incidence of cervical and endometrial cancer on average in the Bryansk region for 2020 shows a decrease in real values in comparison with the forecast data by 20.7% and 10.6%, respectively.

## Figures and Tables

**Table 1:** Ranking of areas within the Bryansk region by the level of radiation, chemical, and combined environmental contamination and the incidence of cervical and endometrial cancer of the female population in 2000–2019 (per 100,000).

No	Cities and Districts of the Bryansk Region	Main Gaseous Air Pollutants					Contamination Density, kBq/m^2^		Incidence of Cervical Cancer, M ± SEM	Incidence of Endometrium Cancer, M ± SEM
		Total	Of These:							
			VOCs	NO_x_	SO_2_	CO	^137^Cs	^90^Sr		
		Gross Emissions of Gaseous Pollutants per Area, g/m^2^								
**Environmentally safe areas (control)**										
1	Rognedinsky *(n = 3416)*	13	0	6	0	7	21.7	0.8	24.7 ± 7.1	21.5 ± 5.5
	Suzemsky *(n = 7845)*	28	5	9	1	13	18.6	2.5	28.1 ± 4.3	46.6 ± 7.5
	Mglinsky *(n = 8698)*	31	6	6	2	17	6.6	0.6	16.4 ± 4.3	23.3 ± 4.7
	Kletnyansky *(n = 8990)*	47	27	5	5	10	5.4	0.5	16.6 ± 3.5	25.2 ± 4.4
	Navlinsky *(n = 12,468)*	54	12	13	4	25	18.9	0.8	23.0 ± 3.0	35.5 ± 4.2
	Dubrovsky *(n = 9087)*	56	13	17	0.4	26	7.2	0.4	12.9 ± 3.1	25.0 ± 5.0
	Brasovsky *(n = 9423)*	64	10	19	6	29	25.2	0.4	18.6 ± 4.3	37.1 ± 4.3
	Sevsky *(n = 7581)*	68	20	10	24	14	18.9	1.4	22.1 ± 5.3	35.3 ± 4.5
	Komarichsky *(n = 8086)*	99	25	19	9	46	27.1	1.0	21.0 ± 3.7	30.6 ± 4.5
	Karachevsky *(n = 16,442)*	115	29	34	1	51	13.9	0.8	18.5 ± 3.4	37.7 ± 5.0
	Surazhsky *(n = 10,894)*	128	35	35	6	52	8.2	0.4	18.9 ± 4.0	27.8 ± 3.9
	**Average value**	**64**	**17**	**16**	**5.3**	**26**	**16**	**0.9**	19.7 ± 1.7 * ***−14.9%***	32.3 ± 3.0 * ***−8.7%***
**Areas of chemical pollution**										
2	Pogarsky *(n = 13,398)*	123	65	22	4	32	29.9	1.1	18.9 ± 3.4	45.6 ± 6.4
	Zhiryatinsky *(n = 3274)*	156	104	16	1	35	5.4	0.8	19.5 ± 5.1	32.6 ± 6.3
	Zhukovsky *(n = 16,428)*	195	22	53	40	80	6.6	0.8	22.4 ± 3.1	28.3 ± 3.1
	Trubchevsky *(n = 16,659)*	275	88	27	2	158	23.6	0.8	19.1 ± 2.7	38.0 ± 5.1
	Pochepsky *(n = 18,827)*	365	223	33	3	106	5.4	0.5	15.8 ± 2.2	31.9 ± 4.2
	Unechsky *(n = 18,519)*	559	292	58	32	177	7.2	0.8	25.0 ± 3.4	31.1 ± 3.0
	Vygonichsky *(n = 9155)*	858	749	37	2	70	9.5	0.4	22.6 ± 3.3	12.7 ± 3.9
	Bryansky *(n = 24,737)*	959	813	47	13	86	5.7	0.4	18.2 ± 2.5	32.6 ± 3.8
	Town Seltso *(n = 8140)*	5209	773	2405	97	1934	4.4	0.8	24.2 ± 6.3	33.8 ± 5.6
	Dyatkovsky *(n = 33,907)*	8045	339	3760	1139	2807	38.4	1.1	20.8 ± 3.1	35.7 ± 3.9
	City Bryansk *(n = 202,954)*	32190	5217	10886	2617	13470	8.8	5.9	17.2 ± 1.4	41.6 ± 3.1
	**Average value**	**4449**	**790**	**1577**	**359**	**1723**	**13**	**1.2**	18.6 ± 1.6 * ***−19.7%***	38.2 ± 3.0 * ***+8.0%***
**Areas of radioactive contamination**										
3	Krasnogorsky *(n = 6273)*	15	1	5	0	9	303.4	9.3	12.6 ± 3.6	51.3 ± 7.2
	Gordeevsky *(n = 5197)*	28	2	11	0.2	15	328.6	5.0	21.8 ± 5.2	31.2 ± 6.6
	Zlynkovsky *(n = 5654)*	38	5	11	4	18	412.4	16.3	18.6 ± 5.1	26.7 ± 4.4
	Novozybkovsky *(n = 5558)*	51	10	0	0	41	460.6	8.4	23.3 ± 5.8	18.2 ± 4.5
	Klimovsky *(n = 13,731)*	72	16	8	15	33	139.6	6.4	20.8 ± 4.0	38.6 ± 7.4
	Klintsovsky *(n = 8920)*	169	17	70	2	80	194.4	4.7	19.0 ± 3.0	18.6 ± 3.1
	**Average value**	**62**	**8.5**	**18**	**3.5**	**33**	**307**	**8.4**	19.3 ± 2.7 * ***−16.7%***	32.5 ± 3.8 * ***−8.1%***
**Areas of combined radiation-chemical contamination**										
4	Starodubsky *(n = 18,247)*	392	316	24	9	43	45.4	1.4	14.7 ± 2.1	26.1 ± 3.0
	City Klintsy *(n = 32,128)*	7264	2059	2616	139	2450	195.6	3.0	20.9 ± 2.1	39.9 ± 3.3
	Sity Novozybkov *(n = 18,294)*	7422	1778	2159	406	3079	456.5	9.7	23.6 ± 2.7	42.4 ± 4.7
	**Average value**	**5026**	**1384**	**1600**	**185**	**1857**	**233**	**4.7**	20.0 ± 1.7 * ***−13.6%***	36.9 ± 2.7 * ***+4.3%***

Note: *. Difference (%) from the all-Russian incidence (2000–2019). Significance level while checking the hypothesis about differences of incidence of cervical and endometrial cancer according to the Wilcoxon rank-sum (Mann–Whitney) test: 1. In environmentally safe areas and areas of chemical (*p* = 0.74; 0.42), radioactive (*p* = 0.96; 0.84), and combined contamination (*p* = 0.94; 0.24); chemical and radioactive (*p* = 0.72; 0.48), chemical and combined (*p* = 0.94; 0.59), radioactive and combined (*p* = 0.61; 0.44) contamination. 2. In environmentally safe areas (*p* = 0.08; 0.37), areas of chemical pollution (*p* = 0.02; 0.55), radioactive (*p* = 0.09; 0.23), and combined contamination (*p* = 0.07; 0.65) with the all-Russian values. n = average sample size for the female population over 18 years old for 2000–2019.

**Table 2:** Dynamics of the incidence of cervical and endometrial cancer in environmentally different areas of the Bryansk region in 2000–2020 (absolute values).

Years	Areas *							
	*CP*	*RC*	CC	*ES*	*CP*	*RC*	CC	*ES*
	Cervical Cancer, abs.				Endometrial Cancer, abs.			
2000	65	16	14	10	110	12	19	24
2001	36	6	13	16	88	9	23	30
2002	49	2	7	17	112	14	26	21
2003	53	7	14	18	126	5	16	22
2004	29	4	6	11	91	17	10	23
2005	36	1	12	13	58	5	14	13
2006	29	4	4	14	86	5	19	12
2007	49	4	14	18	93	9	22	24
2008	66	8	15	10	93	10	27	29
2009	72	4	19	18	164	17	24	32
2010	67	13	15	24	121	18	26	31
2011	75	15	15	26	162	11	21	39
2012	77	8	12	27	156	14	40	50
2013	82	6	7	21	173	19	34	56
2014	96	8	19	24	179	11	30	36
2015	83	8	9	20	193	15	33	42
2016	90	10	21	31	201	30	30	40
2017	88	13	19	22	189	20	24	45
2018	108	17	18	23	186	22	36	38
2019	99	15	20	33	197	21	30	44
2020	84	13	15	26	182	23	44	47

* Areas: CP—chemical pollution; RC—radioactive contamination; CC—combined contamination; ES—environmentally safe.

**Table 3:** Dynamics of the frequency of incidence of cervical and endometrial cancer in environmentally different areas of the Bryansk region in 2000–2020 (per 100,000).

Years	Areas *							
	*CP*	*RC*	CC	*ES*	*CP*	*RC*	CC	*ES*
	Cervical Cancer, per 100,000				Endometrial Cancer, per 100,000			
2000	17.5	30.0	19.7	8.8	29.5	22.5	26.7	21.2
2001	9.6	11.5	18.4	14.3	23.6	17.2	32.6	26.9
2002	13.1	3.9	10.0	15.4	29.9	27.4	37.0	19.0
2003	14.2	14.0	19.8	16.3	33.7	10.0	22.6	20.0
2004	7.8	8.2	8.5	10.1	24.3	34.7	14.2	21.1
2005	9.6	2.1	17.0	12.0	15.5	10.3	19.8	12.0
2006	7.8	8.4	5.7	13.1	23.1	10.5	27.1	11.2
2007	13.2	8.5	20.0	16.9	25.0	19.2	31.4	22.6
2008	17.8	17.3	21.5	9.4	25.0	21.7	38.7	27.4
2009	19.4	8.8	27.3	17.1	44.2	37.2	34.5	30.4
2010	18.1	28.8	21.7	22.9	32.7	39.9	37.6	29.6
2011	20.3	33.7	22.1	25.3	43.9	24.7	30.9	38.0
2012	21.0	18.4	17.8	26.7	42.5	32.2	59.4	49.4
2013	22.5	4.2	10.4	21.0	47.5	44.9	50.6	56.1
2014	26.6	19.1	28.3	24.6	49.7	26.2	44.7	36.8
2015	23.2	19.5	13.5	20.8	53.9	36.6	49.3	43.7
2016	25.6	24.8	31.5	32.8	57.1	74.3	45.0	42.3
2017	24.9	32.8	28.6	23.6	53.6	50.4	36.1	48.3
2018	30.8	43.5	27.3	25.2	53.1	56.2	54.6	41.6
2019	28.5	38.9	30.6	36.7	56.7	54.4	45.9	49.0
2020	24.4	34.0	21.5	29.2	52.8	60.2	63.1	52.8

* Areas: CP—chemical pollution; RC—radioactive contamination; CC—combined contamination; ES—environmentally safe.

**Table 4:** Relative risk (RR) of incidence of cervical cancer in environmentally different areas of the Bryansk region in 2000–2019.

Areas	Female Population of the Bryansk Region in 2000–2019	Got Sick, abs.	Did not Get Sick, abs.	RR (95% CI)
Chemical, radioactive and combined contamination (total)	9,599,974	1791	9,598,183	0.97 (0.87–1.08)
Environmentally safe	2,058,551	396	2,058,155	
Chemical pollution	7,319,942	1349	7,318,593	0.96 (0.86–1.07)
Environmentally safe	2,058,551	396	2,058,155	
Radioactive contamination	906,651	169	906,482	0.97 (0.81–1.16)
Environmentally safe	2,058,551	396	2,058,155	
Combine contamination	1,373,381	273	1,373,108	1.03 (0.89–1.21)
Environmentally safe	2,058,551	396	2,058,155	
Radioactive contamination	906,651	169	906,482	1.01 (0.86–1.19)
Chemical pollution	7,319,942	1349	7,318,593	
Combine contamination	1,373,381	273	1,373,108	1.08 (0.95–1.23)
Chemical pollution	7,319,942	1349	7,318,593	
Combine contamination	1,373,381	273	1,373,108	1.07 (0.88–1.30)
Radioactive contamination	906,651	169	906,482	

**Table 5:** Relative risk (RR) of incidence of endometrial cancer in environmentally different areas of the Bryansk region in 2000–2019.

Areas	Female Population of the Bryansk Region in 2000–2019	Got Sick, abs.	Did not Get Sick, abs.	RR (95% CI)
Chemical, radioactive and combined contamination (total)	9,599,974	3566	9,596,408	**1.17** **(1.08–1.27)**
Environmentally safe	2,058,551	651	2,057,900	
Chemical pollution	7,319,942	2778	7,317,164	**1.20** **(1.10–1.31)**
Environmentally safe	2,058,551	651	2,057,900	
Radioactive contamination	906,651	284	906,367	0.99 (0.86–1.14)
Environmentally safe	2,058,551	651	2,057,900	
Combine contamination	1,373,381	504	1,372,877	**1.16** **(1.03–1.30)**
Environmentally safe	2,058,551	651	2,057,900	
Radioactive contamination	906,651	284	906,367	**0.83** **(0.73–0.93)**
Chemical pollution	7,319,942	2778	7,317,164	
Combine contamination	1,373,381	504	1,372,877	0.97 (0.88–1.06)
Chemical pollution	7,319,942	2778	7,317,164	
Combine contamination	1,373,381	504	1,372,877	**1.17** **(1.01–1.35)**
Radioactive contamination	906,651	284	906,367	

**Table 6:** Correlation analysis of the incidence of cervical and endometrial cancer in cities and districts of the Bryansk region by the level of radioactive contamination and chemical pollution of the environment (2000–2019).

Cities and Districts of the Bryansk Region	Main Gaseous Air Pollutants					Contamination Density, kBq/m^2^		Incidence of Cervical Cancer (per 100,000)	Incidence of Endometrium Cancer (per 100,000)
	Total	Of These:							
		VOCs	NO_x_	VOCs	CO	^137^Cs	^90^Sr		
	Gross Emissions of Gaseous Pollutants per Area, g/m^2^								
Rognedinsky	13	0	6	0	7	21.7	0.8	24.7	21.5
Suzemsky	28	5	9	1	13	18.6	2.5	28.1	46.6
Mglinsky	31	6	6	2	17	6.6	0.6	16.4	23.3
Kletnyansky	47	27	5	5	10	5.4	0.5	16.6	25.2
Navlinsky	54	12	13	4	25	18.9	0.8	23.0	35.5
Dubrovsky	56	13	17	0,4	26	7.2	0.4	12.9	25.0
Brasovsky	64	10	19	6	29	25.2	0.4	18.6	37.1
Sevsky	68	20	10	24	14	18.9	1.4	22.1	35.3
Komarichsky	99	25	19	9	46	27.1	1.0	21.0	30.6
Karachevsky	115	29	34	1	51	13.9	0.8	18.5	37.7
Surazhsky	128	35	35	6	52	8.2	0.4	18.9	27.8
Pogarsky	123	65	22	4	32	29.9	1.1	18.9	45.6
Zhiryatinsky	156	104	16	1	35	5.4	0.8	19.5	32.6
Zhukovsky	195	22	53	40	80	6.6	0.8	22.4	28.3
Trubchevsky	275	88	27	2	158	23.6	0.8	19.1	38.0
Pochepsky	365	223	33	3	106	5.4	0.5	15.8	31.9
Unechsky	559	292	58	32	177	7.2	0.8	25.0	31.1
Vygonichsky	858	749	37	2	70	9.5	0.4	22.6	12.7
Bryansky	959	813	47	13	86	5.7	0.4	18.2	32.6
Town Seltso	5209	773	2405	97	1934	4.4	0.8	24.2	33.8
Dyatkovsky	8045	339	3760	1139	2807	38.4	1.1	20.8	35.7
City Bryansk	32,190	5217	10,886	2617	13,470	8.8	5.9	17.2	41.6
Krasnogorsky	15	1	5	0	9	303.4	9.3	12.6	51.3
Gordeevsky	28	2	11	0,2	15	328.6	5.0	21.8	31.2
Zlynkovsky	38	5	11	4	18	412.4	16.3	18.6	26.7
Novozybkovsky	51	10	0	0	41	460.6	8.4	23.3	18.2
Klimovsky	72	16	8	15	33	139.6	6.4	20.8	38.6
Klintsovsky	169	17	70	2	80	194.4	4.7	19.0	18.6
Starodubsky	392	316	24	9	43	45.4	1.4	14.7	26.1
City Klintsy	7264	2059	2616	139	2450	195.6	3.0	20.9	39.9
Sity Novozybkov	7422	1778	2159	406	3079	456.5	9.7	23.6	42.4
Correlation coefficients *(ρ)* and levels of their statistical significance *(p)*									
**Cervical cancer**									
–	ρ = 0.05 *p* = 0.79	ρ = −0.01 *p* = 0.95	ρ = 0.09 *p* = 0.64	ρ = 0.13 *p* = 0.50	ρ = 0.11 *p* = 0.57	ρ = 0.13 *p* = 0.50	ρ = 0.19 *p* = 0.31	–	–
**Endometrium cancer**									
–	ρ = 0.19 *p* = 0.32	ρ = 0.20 *p* = 0.28	ρ = 0.18 *p* = 0.32	ρ = 0.30 *p* = 0.10	ρ = 0.16 *p* = 0.38	ρ = 0.18 *p* = 0.35	ρ = 0.34 *p* = 0.06	–	–

## Data Availability

The authors used the depersonalized data of official state statistics by the incidence of cervical and endometrium cancer in woman over 18 years of age for 2000–2020 according to the data of the Bryansk regional oncological dispensary (Cancer registry of the Bryansk region). These data were provided for scientific research without the right to distribute.
